# A Semi-Automatic Magnetic Resonance Imaging Annotation Algorithm Based on Semi-Weakly Supervised Learning

**DOI:** 10.3390/s24123893

**Published:** 2024-06-16

**Authors:** Shaolong Chen, Zhiyong Zhang

**Affiliations:** 1 School of Sino-German Intelligent Manufacturing, Shenzhen City Polytechnic, Shenzhen 518000, China; chenshlong@mail2.sysu.edu.cn; 2 School of Electronics and Communication Engineering, Sun Yat-Sen University, Shenzhen 518000, China

**Keywords:** semi-supervised learning, weakly supervised learning, active learning, magnetic resonance image, semi-automatic annotation

## Abstract

The annotation of magnetic resonance imaging (MRI) images plays an important role in deep learning-based MRI segmentation tasks. Semi-automatic annotation algorithms are helpful for improving the efficiency and reducing the difficulty of MRI image annotation. However, the existing semi-automatic annotation algorithms based on deep learning have poor pre-annotation performance in the case of insufficient segmentation labels. In this paper, we propose a semi-automatic MRI annotation algorithm based on semi-weakly supervised learning. In order to achieve a better pre-annotation performance in the case of insufficient segmentation labels, semi-supervised and weakly supervised learning were introduced, and a semi-weakly supervised learning segmentation algorithm based on sparse labels was proposed. In addition, in order to improve the contribution rate of a single segmentation label to the performance of the pre-annotation model, an iterative annotation strategy based on active learning was designed. The experimental results on public MRI datasets show that the proposed algorithm achieved an equivalent pre-annotation performance when the number of segmentation labels was much less than that of the fully supervised learning algorithm, which proves the effectiveness of the proposed algorithm.

## 1. Introduction

In recent years, due to its advantages of a simple design, strong generalization ability and high precision, deep learning has been widely used in medical image processing [1,2,3,4,5,6]. At present, in the research on medical image processing based on deep learning, it is still the mainstream way to train deep learning models with annotated medical images through fully supervised learning [7,8,9,10]. Therefore, annotated medical image datasets are very important for medical image processing based on deep learning. Due to the professionalism of medical images, the annotation of medical images is generally completed by experienced doctors with the help of medical image annotation tools. Convenient and efficient annotation tools can improve the efficiency of medical image annotation and reduce the workload of annotation.

At present, the tools that can annotate medical images are mainly divided into general image annotation tools and medical image annotation tools. By converting medical images into general image formats, the annotation of medical images can also be realized by general image annotation tools. There are many general image annotation tools available, such as VIA [11], Ratsnage [12], fluid annotation [13], LabelMe [14], iVAT [15], Bayesian-CRF [16], etc. Generally, these general image annotation tools cannot directly annotate commonly used medical image formats (such as DICOM, NIFTI, MHD+RAW, ANALYZE, etc.), and the medical images need to be converted into general image formats first, which decreases the efficiency. In addition, in the face of some special requirements of medical image annotation tasks, general image annotation tools may not be applicable. There are few medical image annotation tools that can annotate commonly used medical image formats. These mainly include ITK-SNAP 4.2.0 [17], Image J2 [18], MIPAV 11.2.0 [19], 3D Slicer 5.6.2 [20], etc. These medical image annotation tools all provide semi-automatic annotation methods. However, the pre-annotation of these tools is based on the traditional segmentation algorithm, and the performance is relatively poor and the efficiency of annotation is limited.

In recent years, due to the great achievements of deep learning in the field of medical image segmentation [21,22,23], researchers began to explore semi-automatic annotation based on deep learning [24,25,26,27,28,29]. Pair [30] is a semi-automatic annotation tool based on deep learning developed by Shenzhen University for medical image annotation tasks. It uses a deep learning-based segmentation algorithm to provide pre-annotation, which can effectively improve the annotation efficiency of doctors. Deng et al. [24] introduced active learning into the field of semi-automatic annotation, and selected the most effective data through the uncertainty criterion for doctors to manually annotate, thus improving the contribution rate of a single annotation sample to the performance of the pre-annotation model. Compared with the semi-automatic annotation algorithm based on traditional segmentation algorithms, the semi-automatic annotation algorithm based on deep learning significantly improved the efficiency of annotation. However, most of the existing semi-automatic annotation algorithms based on deep learning use fully supervised learning to train deep learning models, which may lead to poor pre-annotation performance and limited improvement of annotation efficiency when segmentation labels are insufficient.

In this paper, we propose a semi-automatic MRI annotation algorithm based on semi-weakly supervised learning. In order to achieve a better pre-annotation performance in the case of insufficient segmentation labels, semi-supervised and weakly supervised learning are introduced, and a semi-weakly supervised learning segmentation algorithm based on sparse label is proposed. In addition, in order to improve the contribution rate of a single segmentation label to the performance of the pre-annotation model, an iterative annotation strategy based on active learning was designed. The experimental results on the public MRI dataset OAI-ZIB [31] show that the proposed algorithm achieved an equivalent pre-annotation performance when the number of segmentation labels was much less than that of the fully supervised learning algorithm, which proves the effectiveness of the proposed algorithm.

The contributions of this study are as follows:

1. A semi-weakly supervised learning segmentation algorithm based on sparse label is proposed, which uses a few sparse segmentation labels and a large number of bounding box labels into a joint train segmentation network. To the best of our knowledge, this is the first time that semi-weakly supervised learning has been introduced into medical image annotation.
2. An iterative annotation strategy based on active learning is proposed to improve the contribution rate of a single annotation sample to the performance of the pre-annotation model.
3. A sparse annotation strategy for MRI annotation is proposed, and the rationality and effectiveness of the proposed method were verified by experiments.

## 2. Related Work

In recent years, deep learning has achieved great success in the field of medical image segmentation, and some researchers have proposed semi-automatic annotation algorithms based on deep learning. Zhang et al. [32] proposed an interactive image annotation framework based on composite geodesic distance, which was tested on prostate MRI datasets. Compared with traditional interactive annotation methods, the proposed method achieved higher accuracy with fewer interactive annotations and in a shorter time. Li et al. [33] proposed a hybrid active learning framework using interactive annotations for medical image segmentation. Experiments were conducted on four medical image datasets, and the proposed framework achieved high-precision pixel by pixel annotation and generated a model with fewer labels on the data and fewer interactions. Dong et al. [34] proposed a medical image semi-automatic annotation system based on deep learning to address the problems of high workloads and low consistency in free drawing. The system implements pre-annotation based on deep learning models and can be corrected through polygons or free drawing. Chen et al. [35] developed an online semi-automatic annotation system for medical images, which supports annotation modes such as polygons and free drawing. It can provide semi-automatic auxiliary annotation through convolutional neural networks. The system is based on a browser server mode and runs in a browser without any installation. However, these semi-automatic annotation methods use fully supervised learning to train pre-annotation models, which may result in poor pre-annotation performance and limited improvement in annotation efficiency in cases of insufficient segmentation labels.

## 3. Methods

**Figure 1:**
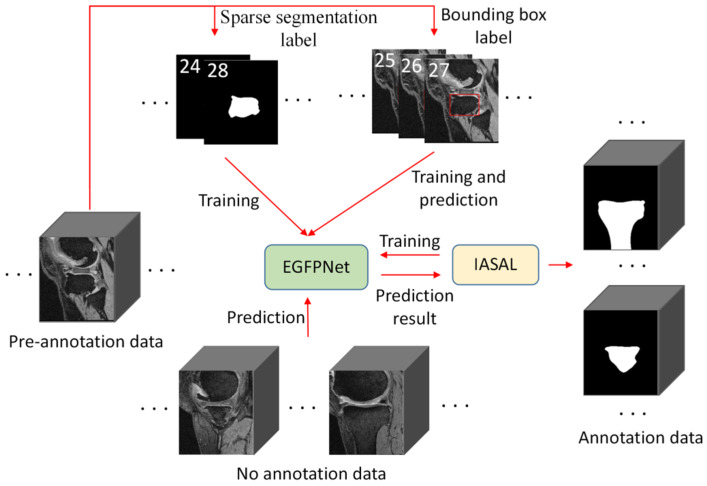
The proposed semi-automatic MRI annotation algorithm based on semi-weakly supervised learning. EGFPNet: edge guidance feature pyramid network. IASAL: iterative annotation strategy based on active learning. A few sparse segmentation labels and a large number of bounding box labels are used to jointly train EGFPNet. EGFPNet and IASAL interactive prediction and training.

The proposed semi-automatic MRI annotation algorithm based on semi-weakly supervised learning is shown in Figure 1, and mainly includes the following three parts.

(1) An edge guidance feature pyramid network (EGFPNet). EGFPNet [36] is a network proposed by the authors, which mainly introduces edge information to improve the quality of edge segmentation. In this study, we used EGFPNet as the base network because the quality of edge segmentation is important for improving the efficiency of semi-automatic annotation, and EGFPNet can improve the performance of tissue edge segmentation.
(2) Semi-weakly supervised learning segmentation algorithm based on sparse labels (SWSSL). A pre-annotation model is obtained by training the EGFPNet with a few sparse segmentation labels and a large number of bounding box labels.
(3) Iterative annotation strategy based on active learning (IASAL). The pre-annotation model predicts all the unannotated data to obtain the prediction results. Considering the similarity of anatomical structures between adjacent MRI slices and the sample difficulty, the appropriate prediction results are selected based on active learning and correction by doctors. The corrected prediction results are used to train the pre-annotation model. Iterative model updating and annotation are performed until all unannotated data has been annotated.

In Section 3.1, we introduce the semi-weakly supervised learning segmentation algorithm based on sparse labels. In Section 3.2, we introduce the iterative annotation strategy based on active learning.

### 3.1. Semi-Weakly Supervised Learning Segmentation Algorithm Based on Sparse Labels

**Figure 2:**
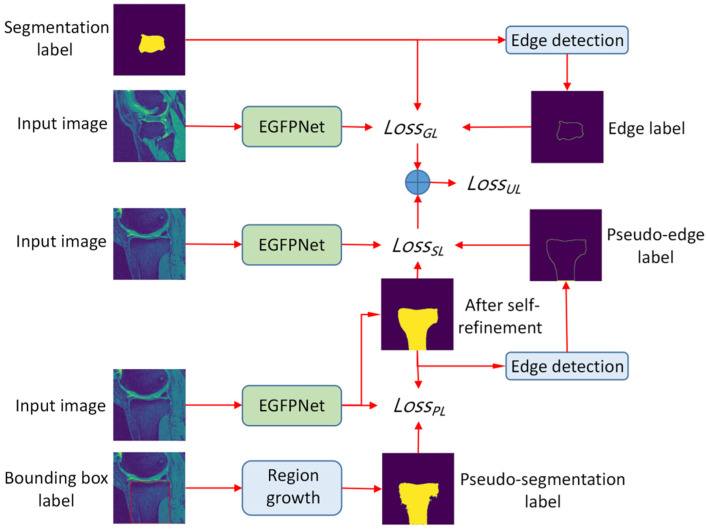
The proposed semi-weakly supervised learning segmentation algorithm based on sparse labels. ${Loss}_{GL}$: the loss function for training the EGFPNet based on sparse segmentation labels and the corresponding edge labels. *Loss_PL_*: the loss function for training the EGFPNet based on pseudo-segmentation labels. *Loss_SL_*: the loss function for training the EGFPNet based on pseudo-segmentation labels and the corresponding pseudo-edge labels. *Loss_UL_*: the loss function of the joint training of the EGFPNet. The function of edge detection is to convert segmentation labels into edge labels, providing edge labels for the training of EGFPNet.

The proposed semi-weakly supervised learning segmentation algorithm based on sparse labels (SWSSL) is shown in Figure 2, and mainly includes the following three parts.

(1) Training EGFPNet based on sparse segmentation labels. A few sparse segmentation labels are input to the edge detection algorithm to generate the corresponding edge labels, and to the EGFPNet for training.
(2) Training EGFPNet based on bounding box labels. The region growth algorithm generates pseudo-segmentation labels based on a large number of boundary box labels. The pseudo-segmentation labels train the EGFPNet, the pseudo-segmentation labels are updated by self-refinement. Because there is a certain gap between the pseudo-edge label generated by the pseudo-segmentation labels and the actual edge label, the pseudo-edge label does not participate in the training at this stage.
(3) Joint training of EGFPNet. A few sparse segmentation labels and the corresponding edge labels, and a large number of pseudo-segmentation labels and the corresponding pseudo-edge labels are used to jointly train the EGFPNet.

#### 3.1.1. Training EGFPNet Based on Sparse Segmentation Labels

**Figure 3:**
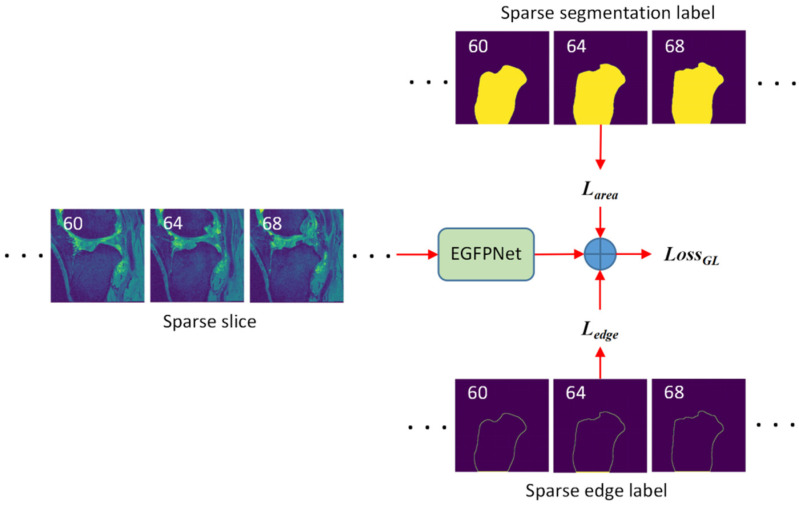
Training EGFPNet based on sparse segmentation label. $L_{area}$: the loss function for training the EGFPNet based on segmentation labels. *L_edge_*: the loss function for training the EGFPNet based on edge labels. *Loss_GL_*: the loss function for training the EGFPNet based on sparse segmentation labels and the corresponding edge labels.

The training of the EGFPNet based on sparse segmentation labels is shown in Figure 3. As shown in Figure 3, for each 3D MRI, sparse segmentation labels are annotated after a certain number of slices ($S_{int}=3$). Sparse edge labels are automatically obtained by sparse segmentation labels through the edge detection algorithm. The main idea of edge detection algorithm is as follows: at position (x,y), if the segmentation label $G_{x,\;y}$ itself and its adjacent 8 pixels are all foreground pixels, then the segmentation label $G_{x,\;y}$ at position (x,y) is not an edge. If the segmentation label $G_{x,\;y}$ itself is a foreground pixel and the adjacent 8 pixels have background points, then the segmentation label $G_{x,\;y}$ at position (x,y) is an edge.

The input to the EGFPNet is 2D MRI slices, and the network is training using segmentation labels and edge labels. For the segmentation label loss function $L_{area}$, consider the combination of binary cross entropy loss and dice loss,

(1) $$L_{area}=L_{BCE}+L_{DICE}$$

Here,

(2) $$L_{BCE}=\sum_{x,\;y}\left(G_{x,\;y}log(Q_{x,\;y})+{(1-G}_{x,\;y})log(1-Q_{x,\;y})\right)$$

(3) $$L_{DICE}=1-\frac{2\sum_{x,\;y}\left(G_{x,\;y}Q_{x,\;y}\right)}{\sum_{x,\;y}\left(G_{x,\;y}+Q_{x,\;y}\right)}$$

where $L_{BCE}$ and $L_{DICE}$ represent the binary cross entropy loss and dice loss, respectively. $G_{x,\;y}\in \left\{0,1\right\}$ is the segmentation label at position (x,y), and $Q_{x,\;y}\in \left[0,1\right]$ is the segmentation prediction result at position (x, y).

For the edge label loss function $L_{edge}$, consider the weight binary cross entropy loss,

(4) $$L_{edge}=\sum_{x,\;y}\left({w_{0}E}_{x,\;y}log(S_{x,\;y})+w_{1}{(1-E}_{x,\;y})log(1-S_{x,\;y})\right)$$

Here,

(5) $$w_{0}=\frac{\sum_{x,\;y}E_{x,\;y}}{WH}$$

(6) $$w_{1}=1-w_{0}$$

where $E_{x,\;y}\in \left\{0,\;1\right\}$ is the edge label at position (x, y), and $S_{x,\;y}\in \left[0,\;1\right]$ is the edge prediction result at position (x, y). $w_{0}$ and $w_{1}$ represent the weights of label 0 and label 1, respectively. W and H represent the width and height of the label, respectively.

The total loss function of the sparse segmentation label and corresponding edge label ${Loss}_{GL}$ is

(7) $${Loss}_{GL}=L_{area}+L_{edge}$$

#### 3.1.2. Training EGFPNet Based on Bounding Box Labels

**Figure 4:**
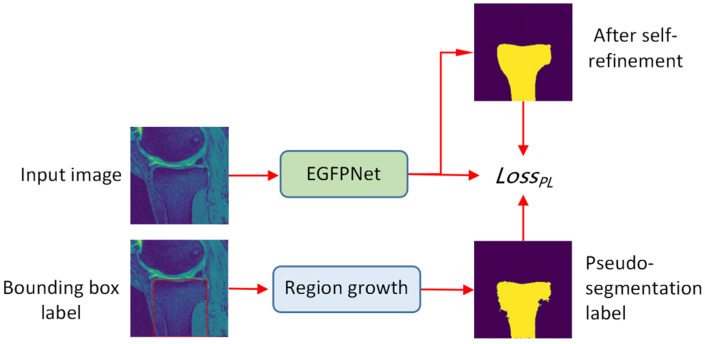
Training EGFPNet based on bounding box labels. ${Loss}_{PL}$: the loss function for training the EGFPNet based on pseudo-segmentation labels.

The training of the EGFPNet based on bounding box labels is shown in Figure 4. As shown in Figure 4, the main steps are as follows:

(1) The region growing algorithm generates pseudo-segmentation labels. The selection of seed points of different tissues is inconsistent in the region growth. For bone tissue, according to prior knowledge, the center point of the boundary box is directly used as the seed point. For cartilage tissue, the canny algorithm detects the edge, and the midpoint of the thickest part of the longitudinal edge is used as the seed point. The stop rule is that the area of the growing area is greater than or equal to the boundary box area (0.8 for bone tissue) and the area of the growing area is greater than or equal to the boundary box area (0.6 for cartilage tissue). Due to the significant difference between the pseudo-segmentation labels generated by bounding box labels and the real segmentation labels, the region growing algorithm generates pseudo-segmentation labels based on a large number of bounding box labels.
(2) The generated pseudo-segmentation labels are used to train the EGFPNet. Because there is a certain gap between the pseudo-edge labels generated by pseudo-segmentation labels and the actual edge labels, pseudo-edge labels do not participate in training at this stage. Therefore, the edge branches of the network only used to make predictions and do not participate in training.
(3) Pseudo-segmentation label self-refinement. Using the predicted results of the EGFPNet as pseudo-segmentation labels, the network parameters and pseudo-segmentation labels are iteratively updated. The prediction results of the EGFPNet are used as pseudo-segmentation labels to iteratively update the network parameters and pseudo-segmentation labels.

For the pseudo-segmentation label loss function ${Loss}_{PL}$, consider the combination of binary cross entropy loss and dice loss,

(8) $${Loss}_{PL}=L_{P\_BCE}+L_{P\_DICE}$$

Here,

(9) $$L_{P\_BCE}=\sum_{x,\;y}\left(P_{x,\;y}log(T_{x,\;y})+{(1-P}_{x,\;y})log(1-T_{x,\;y})\right)$$

(10) $$L_{P\_DICE}=1-\frac{2\sum_{x,\;y}\left(P_{x,\;y}T_{x,\;y}\right)}{\sum_{x,\;y}\left(P_{x,\;y}+T_{x,\;y}\right)}$$

where $L_{P\_BCE}$ and $L_{P\_DICE}$ represent the binary cross entropy loss and dice loss, respectively. $P_{x,\;y}\in \left\{0,\;1\right\}$ is the pseudo-segmentation label at position (x, y), and $T_{x,\;y}\in \left[0,\;1\right]$ is the segmentation prediction result at position (x, y).

#### 3.1.3. Joint Training of EGFPNet

In the early stage of training, a large number of pseudo-segmentation labels are used to guide the EGFPNet to learn high-level semantic information such as the location of the tissue. With the increase of training times, the segmentation label is used to guide the EGFPNet to gradually learn low-level semantic information such as the edge of the tissue, so as to suppress the false guidance of using the pseudo-labels as supervision information. The loss function of joint training

(11) $${Loss}_{UL}={\alpha Loss}_{SL}+\beta {Loss}_{GL}$$

where ${Loss}_{GL}$ represents the loss function of training the EGFPNet based on sparse segmentation labels and the corresponding edge labels. ${Loss}_{SL}$ represents the loss function for training the EGFPNet based on the pseudo-segmentation labels and the corresponding pseudo-edge labels. α and β represent the weights of the loss functions ${Loss}_{SL}$ and ${Loss}_{GL}$, respectively.

(12) $${Loss}_{SL}={Loss}_{PL}+L_{P\_edge}$$

(13) $$\alpha =max(\frac{E_{thres}-E_{cur}}{E_{thres}},\;0)$$

(14) $$\beta =min(\frac{E_{cur}}{E_{thres}},\;1)$$

where $E_{thres}$ is a threshold, representing the number of rounds in which the weight coefficient changes. $E_{cur}$ is the current number of training rounds. $L_{P\_edge}$ represents the pseudo-edge label loss function.

(15) $$L_{P\_edge}=\sum_{x,\;y}\left({w_{P0}F}_{x,\;y}log(C_{x,\;y})+w_{P1}{(1-F}_{x,\;y})log(1-C_{x,\;y})\right)$$

Here,

(16) $$w_{P0}=\frac{\sum_{x,\;y}F_{x,\;y}}{WH}$$

(17) $$w_{P1}=1-w_{P0}$$

where $F_{x,\;y}\in \left\{0,\;1\right\}$ is the pseudo-edge label at position (x, y), and $C_{x,\;y}\in \left[0,\;1\right]$ is the edge prediction result at position (x, y). $w_{P0}$ and $w_{P1}$ represent the weight of label 0 and label 1, respectively. W and H indicate the width and height of the label, respectively. The joint training of the EGFPNet is shown in Algorithm 1 ($E_{total}$ represents the total number of training rounds).
**Algorithm 1** Joint training EGFPNet$\mathrm{Input}:\;2D\;\mathrm{MRI}\;\mathrm{slice},\;\mathrm{pseudo-segmentation}\;\mathrm{label}\;P_{x,\;y}$$,\;\mathrm{segmentation}\;\mathrm{label}\;G_{x,\;y}$$,\;\mathrm{threshold}\;E_{thres}$;Output: EGFPNet Q();Initializes the parameters of Q();$\mathrm{For}\;E_{cur}\leq E_{total}$: $\;\;\;\;\;\mathrm{Pseudo-segmentation}\;\mathrm{label}\;P_{x,\;y}$$\;\mathrm{and}\;\mathrm{corresponding}\;\mathrm{pseudo-edge}\;\mathrm{label}\;F_{x,\;y}$ guide Q() training;$\;\;\;\;\;\mathrm{Segmentation}\;\mathrm{label}\;G_{x,\;y}$$\;\mathrm{and}\;\mathrm{corresponding}\;\mathrm{edge}\;\mathrm{label}\;E_{x,\;y}$ guide Q() training;End for

### 3.2. Iterative Annotation Strategy Based on Active Learning

**Figure 5:**
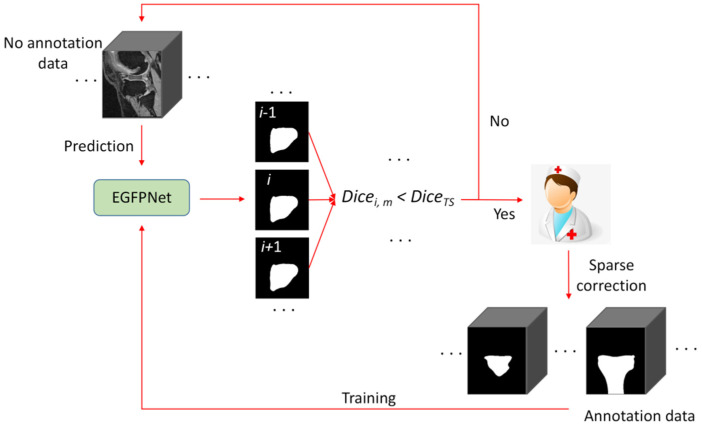
The proposed iterative annotation strategy based on active learning. *Dice_TS_* ∈ [0, 1]; as the number of iteration annotation rounds increases, the threshold *Dice_TS_* increases linearly to 1.

In the stage of iterative model updating and annotation, considering the anatomical structure similarity between adjacent MRI slices and sample difficulty, an iterative annotation strategy based on active learning (IASAL) was designed, as shown in Figure 5. The main steps of IASAL are as follows:

(1) The pre-annotation model predicts all unannotated data and obtains prediction results.

(2) The prediction results of slice *i* in the case $U_{i}$ (*i* = 1, 2, 3, …, *Nu*) are used to calculate the average dice coefficient ${Dice}_{i,\;m}$ along with the prediction results of adjacent slices or segmentation labels. The added slice is to be corrected if ${Dice}_{i,\;m}$ is smaller than the threshold ${Dice}_{TS}$:

(18) $${Dice}_{i,\;m}=\left\{\begin{array}{c} Dice(U_{i},\;U_{i+1}),\;\;\;\;\;\;\;\;\;\;\;\;\;\;\;\;\;\;\;\;\;\;\;\;\;\;\;\;\;\;\;\;\;\;\;\;\;\;\;\;\;\;i=1\;\;\;\;\;\;\;\;\;\;\;\;\;\;\;\;\;\;\;\;\;\;\;\;\;\;\;\;\\ (Dice(U_{i-1},\;U_{i})+Dice(U_{i},\;U_{i+1}))/2,i=2,\;3,\;\ldots ,\;Nu-1\;\\ Dice(U_{i-1},\;U_{i}),\;\;\;\;\;\;\;\;\;\;\;\;\;\;\;\;\;\;\;\;\;\;\;\;\;\;\;\;\;\;\;\;\;\;\;\;\;\;\;\;\;i=Nu\;\;\;\;\;\;\;\;\;\;\;\;\;\;\;\;\;\;\;\;\;\;\;\;\; \end{array}\right.$$

where Dice() indicates the dice coefficient calculation.

(3) The slices to be corrected in each case should be manually sparsely corrected by the doctor (that is, a certain number of slices should be corrected at intervals, and gradually reduced to 0 with the increase in the number of iterative annotation rounds).

(4) All slices after the sparse correction are used to train the pre-annotation model.

(5) Repeat (1), (2), (3) and (4) until all slices are annotated.

## 4. Experiments

### 4.1. Datasets, Pre-Processing, Implementation Details and Evaluation Metrics

#### 4.1.1. Datasets and Pre-Processing

In our experiments, we used a public MRI dataset: OAI-ZIB [31]. All knee MRI images in the OAI-ZIB dataset were from the osteoarthritis initiative, a public database, with a total of 507 cases. The MRI sequences were double-echo steady-state sequences with manually annotated tibia bone (TB), tibial cartilage (TC), femur bone (FB) and femoral cartilage (FC). The MRI image size of each case was 160 × 384 × 384. In the sagittal position, it consisted of 160 slices (2D images) with a size of 384 × 384.

The OAI-ZIB dataset was divided into a training set (70 cases), a validation set (15 cases) and a test set (422 cases). The MRI images were standardized (0–1 standardization) for each case before model training, validation and testing.

#### 4.1.2. Implementation Details

The method in this study is implemented in PyTorch and was run on four RTX 3090 cards. The Adam optimizer was used for training, the batch size was 8 to 32, momentum was 0.9, learning rate was 5 × 10^−4^, weight decay was 1 × 10^−4^, maximum number of iterations was 1000 and the early stop was set to 20.

#### 4.1.3. Evaluation Metrics

We measure the accuracy of segmentation by the dice score (Dice),

(19) $$Dice=\frac{2(A\cap B)}{A\cup B}$$

where A and B represent the prediction result and ground truth, respectively.

### 4.2. Ablation Experiments and Analyses

In order to analyze the components of the proposed algorithm, experiments were conducted on TB and TC.

#### 4.2.1. Effect of Sparse Pre-Annotation Interval on the Performance of Pre-Annotation Model

**Figure 6:**
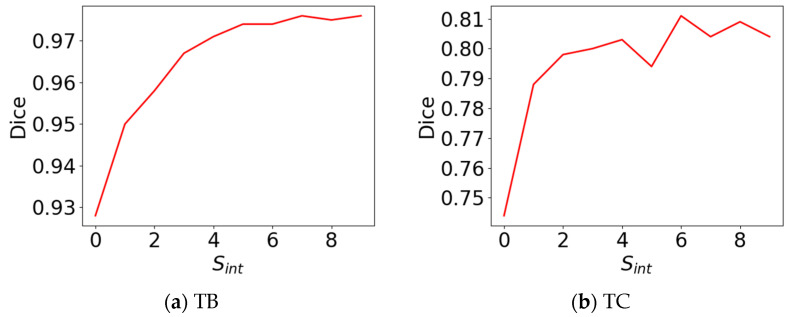
The curve of dice with sparse pre-annotation interval $S_{int}$. TB: tibia bone; TC: tibial cartilage.

In order to analyze the effect of the sparse pre-annotation interval *S_int_* on the performance of the pre-annotation model using the training set, the total number of training slices was kept unchanged (320 slices), and different sparse pre-annotation interval *S_int_* values were selected to train the pre-annotation model, and the performance was evaluated using the test set. The sparse pre-annotation interval *S_int_* was set as follows: (1) $S_{int}$= 0 (no interval, that is, intensive annotation); (2) $S_{int}$= 1; (3) $S_{int}$= 2; (4) $S_{int}$= 3; (5) $S_{int}$= 4; (6) $S_{int}$= 5; (7) $S_{int}$= 6; (8) $S_{int}$ = 7; (9) $S_{int}$= 8; and (10) $S_{int}$= 9. Table 1 shows the effect of the sparse pre-annotation interval $S_{int}$ on the performance of the pre-annotation model. As shown in Table 1, the dice of the pre-annotation model on all tissues increased with an increase in the interval $S_{int}$. However, as the interval $S_{int}$ continued to increase, the dice no longer rose and entered a plateau fluctuation period. This may be because the anatomical structures of adjacent MRI slices are similar. Appropriately increasing the annotation interval is conducive to reducing the information redundancy between annotated slices and improving the contribution rate of single segmentation labels to the performance of the pre-annotation model. However, when the interval increased to a certain extent, the similarity between adjacent slices was very low. Therefore, increasing the interval had little effect for improving the performance of the pre-annotation model. Figure 6 shows the curve of dice with the sparse pre-annotation interval $S_{int}$. As shown in Figure 6, it can be seen more clearly that with the increase in the interval $S_{int}$, the dice of the pre-annotation model on the TB and TC soon entered a plateau fluctuation period after the initial rise. In conclusion, selecting an appropriate sparse pre-annotation interval *S_int_* can improve the performance of the pre-annotation model without increasing the workload of doctors. 

#### 4.2.2. Pseudo-Segmentation Label Self-Refinement

In order to prove the effectiveness of the pseudo-segmentation label self-refinement method proposed in this paper, the performances of the pseudo-segmentation labels generated by different algorithms were compared.

(1) Baseline algorithm. The mask generated by the region growth algorithm was used as pseudo-segmentation labels to train the EGFPNet (labeled ‘Baseline’).
(2) Pseudo-segmentation label self-refinement. The mask generated by the region growth algorithm was used as the pseudo-segmentation labels to train the EGFPNet, and the pseudo-segmentation label used self-refinement (labeled ‘Baseline + PLSR’).

**Figure 7:**
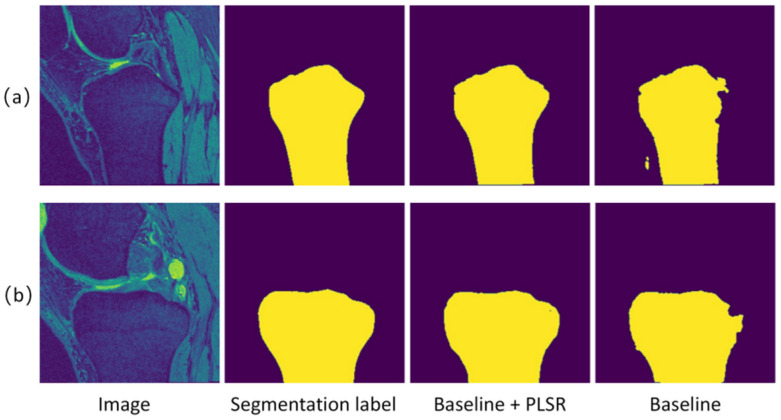
Comparison of pseudo-segmentation label self-refinement (Baseline + PLSR) with baseline (Baseline) on tibia (TB) images. Both (**a**,**b**) represent the results on the TB.

**Figure 8:**
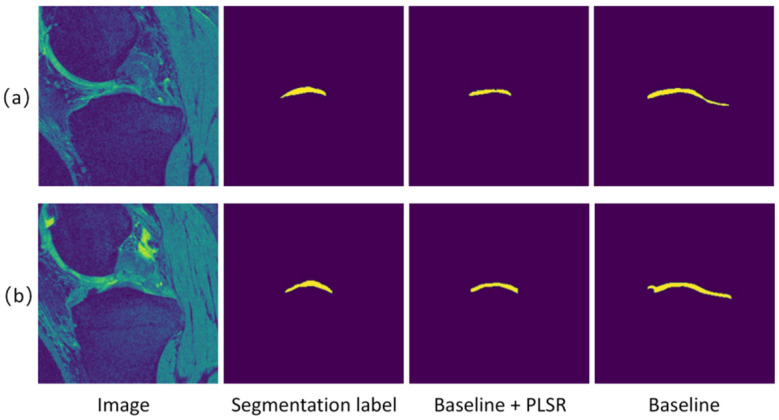
Comparison of pseudo-segmentation label self-refinement (Baseline + PLSR) with baseline (Baseline) on tibial cartilage (TC) images. Both (**a**,**b**) represent the results on the TC.

For all algorithms, the training set of 70 cases was used for training, and the performance was evaluated using the test set. Table 2 shows the results of the pseudo-segmentation label self-refinement compared with the baseline algorithm. As shown in Table 2, compared with the baseline algorithm, the pseudo-segmentation label self-refinement resulted in a significantly improved accuracy of pseudo-segmentation labels. Figure 7 and Figure 8 visualize the segmentation results on TB and TC, respectively. As shown in Figure 7, on the TB, the pseudo-segmentation labels were very close to the true segmentation labels after the pseudo-segmentation label self-refinement. There was still a certain gap between the pseudo-segmentation labels generated by the baseline algorithm and the real segmentation labels in details such as the tissue edge. This is because the pseudo-segmentation labels guiding the training of the baseline algorithm are still very noisy. After many rounds of self-refinement, the pseudo-segmentation labels became closer to the real segmentation labels. As shown in Figure 8, in the TC, compared with the baseline algorithm, the self-refinement pseudo-segmentation labels was closer to the true segmentation labels.

### 4.3. Contrast Experiment

Semi-automatic annotation algorithms based on deep learning are generally divided into two stages: the model pre-training stage and iterative model updating and annotation stage. In order to prove the validity and rationality of the semi-automatic MRI annotation algorithm based on semi-weak supervision proposed in this paper, comparison experiments were set up in the model pre-training stage and iterative model updating and annotation stage.

#### 4.3.1. Model Pre-Training Stage

The main goal of the model pre-training stage is to train the pre-annotation model to have a better performance with fewer segmentation labels or a lower annotation workload. In order to prove the rationality and effectiveness of the semi-weakly supervised learning segmentation algorithm based on sparse labels proposed in this paper, the following comparative experiments were set up:

(1) Fully supervised learning. In the training set, the number of segmentation annotation slices was 320, 1600, 3200 and 4800 (labeled ‘FS’).
(2) Fully supervised learning based on sparse labels. The sparse pre-annotation interval *S_int_* = 3. In the training set, the number of segmentation annotation slices was 320, 1600 and 2800 (all cases have been annotated) (labeled ‘FSS’).
(3) Weakly supervised learning. In the training set, all slices were annotated with bounding box labels (the number of slices was 11,120) (labeled ‘WS’).
(4) Semi-weakly supervised learning segmentation algorithm based on sparse labels. In the training set, the number of segmentation annotation slices was 320, and the remaining slices were annotated with bounding box labels (the number of slices was 10,800) (labeled ‘SWS’).

According to the study of Lin et al. [37], the time spent on segmentation annotation is about 15 times that of the bounding box annotation. Based on this research, this study converted the bounding box annotation workload into an equivalent segmentation annotation workload $S_{eq}$:

(20) $$S_{eq}=S_{s}+S_{bb}/15$$

where $S_{s}$ and $S_{bb}$ represents the number of segmentation annotation slices and bounding box annotation slices, respectively.

Table 3 shows the $S_{eq}$ of each experiment and the results on the test set. As shown in Table 3, the algorithm proposed in this paper has a segmentation annotation workload $S_{eq}$ = 1040, and the segmentation accuracy was slightly lower than that of the fully supervised learning with $S_{eq}$ = 4800, but higher than that of the fully supervised learning with $S_{eq}$ = 3200. Compared with the fully supervised learning, the algorithm proposed in this paper obtained a better segmentation performance with a lower equivalent segmentation annotation workload $S_{eq}$, which proves the rationality of the algorithm proposed in this paper. The algorithm proposed in this paper performed better than the fully supervised learning algorithm based on sparse labels with $S_{eq}$ = 1600 but worse than the fully supervised learning algorithm based on sparse labels with $S_{eq}$ = 2800. Compared with the fully supervised learning algorithm based on sparse labels, the algorithm proposed in this paper obtained a better segmentation performance with a lower equivalent segmentation annotation workload $S_{eq}$. In addition, when the number of equivalent segmentation annotations was slightly higher than that of the weakly supervised learning algorithm, the segmentation performance of the algorithm proposed in this paper in all tissues was significantly better than that of the weakly supervised learning algorithm, which further proves the rationality of the proposed algorithm in this paper. Finally, the tissue segmentation annotation of MRI images is highly specialized and requires expert doctors to perform segmentation annotation, which is costly and also requires a lot of time from doctors. However, the specialization of boundary box annotation in MRI images is relatively low, and it can be annotated by personnel with medical knowledge, which is low-cost and does not require a lot of time from doctors. Therefore, the algorithm proposed in this paper has strong practical application value.

#### 4.3.2. Iterative Model Update and Annotation Stage

The main steps of the iterative model updating and annotation stage are as follows:

(1) The pre-annotation model predicts all unannotated data and obtains the prediction results.
(2) The appropriate prediction results are selected based on the iterative annotation strategy, and doctors correct the prediction results. All slices after correction are used to train the pre-annotation model.
(3) Repeat steps (1) and (2) until all unannotated data are annotated.

It can be seen from the steps of the iterative model updating and annotation stage that after each pre-annotation model update, the accuracy of the prediction results is higher, the doctors have a smaller workload to correct, and the iterative annotation strategy becomes more reasonable. Based on this, the following experiments were set up in this study:

(1) Semi-weakly supervised learning segmentation algorithm based on sparse labels and baseline iterative annotation strategy. The semi-weakly supervised learning segmentation algorithm based on sparse labels was used to obtain the pre-annotation model (the number of segmentation annotation slices were 320, and the remaining slices had bounding box annotations). The baseline iterative annotation strategy (intensive annotation) was use to realize iterative model updating and annotation, and the number of slices annotated by iteration was 800 and 1600, respectively (labeled ‘IA’).
(2) Semi-weakly supervised learning segmentation algorithm based on sparse labels and iterative annotation strategy based on active learning. The semi-weakly supervised learning segmentation algorithm based on sparse labels was used to obtain the pre-annotation model (the number of segmentation annotation slices was 320, and the remaining slices had bounding box annotations). The iterative annotation strategy based on active learning were used to realize iterative model updating and annotation, and the number of slices annotated by iteration was 800 and 1600, respectively (labeled ‘AL’).

Table 4 shows the results of different iterative annotation strategy. As shown in Table 4, when the number of slices annotated by iteration was the same, the iterative annotation strategy based on active learning had a higher segmentation accuracy than the baseline iterative annotation strategy, which proves the validity and rationality of the strategy proposed in this paper.

## 5. Conclusions

In this paper, a semi-automatic MRI annotation algorithm based on semi-weakly supervised learning was proposed to solve the poor performance of existing semi-automatic annotation algorithms based on deep learning have in pre-annotation under the condition of insufficient segmentation labels. Firstly, a semi-weakly supervised learning segmentation algorithm based on sparse label was designed, which uses a few sparse segmentation labels and a large number of bounding box labels to train the EGFPNet. Then, an iterative annotation strategy based on active learning was designed to implement iterative model updating and annotation. The test results on a public MRI dataset showed that the proposed algorithm achieved an equivalent pre-annotation performance when the number of segmentation labels was much less than that of the fully supervised learning algorithm, which proves the effectiveness of the proposed algorithm.

## Figures and Tables

**Table 1:** Effect of sparse pre-annotation interval *S_int_* on the performance of pre-annotation model. TB: tibia bone; TC: tibial cartilage.

	0	1	2	3	4	5	6	7	8	9
TB	0.928	0.950	0.958	0.967	0.971	0.974	0.974	0.976	0.975	0.976
TC	0.744	0.788	0.798	0.800	0.803	0.794	0.811	0.804	0.809	0.804

**Table 2:** Comparison of the pseudo-segmentation label self-refinement with baseline (Baseline + PLSR) with baseline (Baseline) on tibia (TB) and tibial cartilage (TC) images.

	Baseline	Baseline + PLSR
TB	0.948	0.969
TC	0.764	0.785

**Table 3:** Comparison of different segmentation algorithms on tibia (TB), tibial cartilage (TC), femur bone (FB) and femoral cartilage (FC) images. *Seq*: equivalent segmentation annotation workload.

		TB	TC	FB	FC
FS	320	0.928	0.744	0.874	0.496
	1600	0.976	0.808	0.977	0.854
	3200	0.977	0.817	0.980	0.866
	4800	0.980	0.821	0.982	0.868
FSS	320	0.967	0.800	0.952	0.841
	1600	0.977	0.815	0.979	0.865
	2800	0.980	0.820	0.982	0.867
WS	741	0.948	0.764	0.935	0.829
SWS	1040	0.980	0.819	0.981	0.867

**Table 4:** Comparison of results of different iterative annotation strategies on tibia (TB), tibial cartilage (TC), femur bone (FB) and femoral cartilage (FC) images.

		TB	TC	FB	FC
800	IA	0.981	0.820	0.981	0.868
	AL	0.982	0.823	0.982	0.873
1600	IA	0.982	0.822	0.981	0.871
	AL	0.983	0.831	0.983	0.873

## Data Availability

The data used to support the findings of this study are available from the corresponding author upon request.
